# Inhibition of HDAC6 Attenuates Diabetes-Induced Retinal Redox Imbalance and Microangiopathy

**DOI:** 10.3390/antiox9070599

**Published:** 2020-07-09

**Authors:** Hossameldin Abouhish, Menaka C. Thounaojam, Ravirajsinh N. Jadeja, Diana R. Gutsaeva, Folami L. Powell, Mohamed Khriza, Pamela M. Martin, Manuela Bartoli

**Affiliations:** 1 Department of Ophthalmology, Medical College of Georgia, Augusta University, Augusta, GA 30912, USA; habouhish@augusta.edu (H.A.); mthounaojam@augusta.edu (M.C.T.); dgutsaeva@augusta.edu (D.R.G.); 2 Department of Clinical Pharmacology, Faculty of Medicine, Mansoura University, Mansoura 35516, Egypt; mohamedkhriza@gmail.com; 3 Department of Biochemistry and Molecular Biology, Medical College of Georgia, Augusta University, Augusta, GA 30912, USA; rjadeja@augusta.edu (R.N.J.); FPOWELL@augusta.edu (F.L.P.); pmmartin@augusta.edu (P.M.M.)

**Keywords:** diabetic retinopathy, HDAC6, oxidative stress, tubastatin A, retinal endothelial cells, retinal endothelial cell senescence

## Abstract

We investigated the contributing role of the histone deacetylase 6 (HDAC6) to the early stages of diabetic retinopathy (DR). Furthermore, we examined the mechanism of action of HDAC6 in human retinal endothelial cells (HuREC) exposed to glucidic stress. Streptozotocin-induced diabetic rats (STZ-rats), a rat model of type 1 diabetes, were used as model of DR. HDAC6 expression and activity were increased in human diabetic postmortem donors and STZ-rat retinas and were augmented in HuREC exposed to glucidic stress (25 mM glucose). Administration of the HDAC6 specific inhibitor Tubastatin A (TS) (10 mg/kg) prevented retinal microvascular hyperpermeability and up-regulation of inflammatory markers. Furthermore, in STZ-rats, TS decreased the levels of senescence markers and rescued the expression and activity of the histone deacetylase sirtuin 1 (SIRT1), while downregulating the levels of free radicals and of the redox stress markers 4-hydroxynonenal (4-HNE) and nitrotyrosine (NT). The antioxidant effects of TS, consequent to HDAC6 inhibition, were associated with preservation of Nrf2-dependent gene expression and up-regulation of thioredoxin-1 activity. In vitro data, obtained from HuREC, exposed to glucidic stress, largely replicated the in vivo results further confirming the antioxidant effects of HDAC6 inhibition by TS in the diabetic rat retina. In summary, our data implicate HDAC6 activation in mediating hyperglycemia-induced retinal oxidative/nitrative stress leading to retinal microangiopathy and, potentially, DR.

## 1. Introduction

Diabetic retinopathy (DR) is a neurovascular complication of diabetes mellitus and the leading cause of blindness in working age adults [1]. Diabetic retinal microangiopathy significantly contributes to DR pathogenesis and strategies targeting its occurrence and progression are important for preventing vision loss in affected patients [2,3]. 

Hyperglycemia-induced retinal vascular pathology is a multi-step process characterized by retinal endothelial cell dysfunction and death, increased vascular permeability leading to diabetic macular edema (DME), and abnormal retinal neovascularization, as seen in proliferative diabetic retinopathy (PDR) [2,3]. Prolonged hyperglycemia affects the retinal microvasculature by altering multiple molecular pathways involving redox imbalance and induction of pro-inflammatory responses [3]. 

Previously, we showed that hyperglycemia-induced oxidative/nitrative stress accelerates retinal endothelial cell senescence and that this is an important early pathogenic event during the development of diabetic retinal microangiopathy [4]. Retinal oxidative/nitrative stress results from increased production of reactive oxygen and nitrogen species (ROS and RNS, respectively) from cellular and mitochondrial oxidases [5,6] as well as from loss of endogenous antioxidant activities [7,8].

Histone deacetylase 6 (HDAC6) is a class IIb histone deacetylase known to exert important biological functions due to its ability to regulate the acetylation state of nuclear and cytoplasmic proteins [9]. Most known targets of HDAC6 are cytoskeletal proteins, transcription factors [9] and endogenous antioxidants such as peroxiredoxin 1 (Prx1) [10,11]. As a consequence of its multiple biological targets, altered HDAC6 expression and activity is linked to inflammation, oxidative stress and the pathogenesis of a number of neurodegenerative [12] and cardiovascular disorders [13] as well as cancer [14]. 

The potential impact of HDAC6 on diabetic microvascular complications is understudied. Previous studies have shown beneficial effects of HDAC6 inhibition in diabetic kidney disease [15] and diabetic heart disease [16]. Moreover, while little is known on HDAC6 contribution to retinal diabetic disorders, studies have implicated pro-oxidative effects of HDAC6 in animal models of retinal neurodegenerative diseases [17,18], potentially suggesting a role for this histone deacetylase in retinal pathologies involving oxidative stress, such as DR. 

Based on this evidence, we assessed the effects of diabetes on HDAC6 retinal expression and activity in human and experimental DR and we investigated the molecular mechanisms involved in this process in a rat model of type 1 diabetes (streptozotocin-induced diabetic rat = STZ-rat) and in cultures of human retinal microvascular endothelial cells exposed to glucidic stress.

## 2. Materials and Methods 

### 2.1. Human Postmortem Samples

De-identified human postmortem retinal samples were obtained from Georgia Eye Bank, Inc. (Alanta, GA, USA). Retinas were obtained from a total of 8 diabetic and 8 non-diabetic donors that were selected based on DR history or lack of reported ocular pathologies (control). Supplementary Table S1 summarizes the demographic and clinical history information available for all donors. 

### 2.2. Animals and Treatment

All animal experiments strictly adhered to the Statement of the Association for Research in Vision and Ophthalmology (ARVO) for the humane use of laboratory animals for ophthalmological research and to Augusta University approved protocols (#2009-0181). All animals were housed in the vivarium of Augusta University with a 12-h day/night light cycle with light intensity in the room maintained at 130 lux at cage level, and fed ad libitum. Adult male Sprague–Dawley (SD) rats (250−300 g) obtained from Envigo (Indianapolis, IN, USA) were made diabetic by a single intraperitoneal injection of streptozotocin (STZ) (55 mg/kg dissolved in 0.1 mol/L sodium citrate, pH 4.5) (Sigma-Aldrich, St. Louis, MO, USA). Control rats received injections of vehicle alone. Rats with fasting blood glucose ≥250 mg/dL were considered diabetic. A group of STZ-rats was injected intraperitoneally with 10 mg/kg Tubastatin A (TS) (MedChemExpress, Monmouth Junction, NJ, USA) starting two weeks after the STZ injections and continuing every other day for the next six weeks. Control rats received vehicle phosphate-buffered saline (PBS) injection. The diabetic rats were sacrificed by an overdose of anesthesia (ketamine 200 mg/kg and xylazine 60 mg/kg) followed by thoracotomy. Blood glucose levels were measured by ReliOn prime blood glucose monitoring system (Bentonville, AR, USA) and glycated hemoglobin A1c (HbA1c) was measured using A1c Now+ System (PTS Diagnostic, Winter Park, FL, USA). A number of other metabolites were also monitored in diabetic and control rat plasma using a biochemistry panel analyzer (Piccolo Xpress analyzer, Princeton, NJ, USA). Rats metabolic profiles in response to the different treatment protocols are reported in Supplementary Table S2.

### 2.3. Cells and Treatment 

Human retinal endothelial cells (HuREC) were purchased from Cell Systems Corporation (Kirkland, WA, USA) and maintained using complete medium with normal glucose formulation (Cell Systems) at 37 °C and 5% CO_2_ in a humidified atmosphere. All the experiments were carried out using HuREC between passages 3 to 7 and all the tissue culture flasks/plates were pre-coated with attachment factor (Cell Systems). The cells were switched to serum-free medium (Cell Systems) 10 to 12 h before the experiments. To mimic the effects of the hyperglycemia (glucidic stress), HuREC were cultured for 48 h in serum-free medium containing 25 mM d-glucose (high glucose, HG). Similarly, control cells were cultured in serum-free and normal glucose medium (5.5 mM d-glucose, NG). Cells grown in normal glucose media with the addition of l-glucose (5.5 mM d-glucose + 19.5 mM l-glucose, LG) served as an osmotic control. Some of the HuREC were treated with 5 µM TS. To determine TS toxicity towards HuREC, a dose-response curve using MTT cell viability assay (Abcam, Cambridge, MA, country) was conducted following the manufacturer’s instructions. MTT dye absorbance was read using a microplate reader at 540 nm (Supplementary Figure S1).

### 2.4. Histology

Rat eyes from each experimental group were enucleated and embedded in optimal cutting temperature (OCT) mounting medium (Tissue-Tek, Torrance, CA, USA). Samples were then sectioned (10 µm), stained with hematoxylin and eosin (H&E), and examined centrally and on each side (temporal and nasal) of the optic nerve. Retinas were examined using a Zeiss Axioplan-2 microscope (Carl Zeiss, Göttingen, Germany) equipped with a high-resolution camera and processed using imaging Spot Software (version 4.0.2; Diagnostic Instruments, Sterling Heights, MI). Morphometric analysis was conducted to measure total retinal thickness. 

### 2.5. Immunofluorescence

Rat eyes were embedded in OCT mounting medium (Tissue-Tek), frozen on dry ice and then cryostat sectioned. A 4% paraformaldehyde fixative was applied to the slides for 10 min. Slides were incubated overnight at 4 °C with one of the following primary antibodies: Rabbit anti-HDAC6 (Lifespan Biosciences, Seattle, WA, USA) and mouse anti-phosphorylated form of H2A histone family member X (γH2AX) (Cell Signaling Technology, Danvers, MA, USA). Slides were washed three times with 0.1% Triton X-100 in 0.1 M PBS (pH 7.4) followed by a 1-h incubation with one of the following secondary antibodies, all purchased from Molecular Probes-Life Technologies (Grand Island, NY, USA): goat anti-rabbit IgG-conjugated Alexa Fluor 488, goat anti-mouse IgG-conjugated Alexa Fluor 488. Slides were mounted using Fluoroshield mounting medium containing 4′,6-diamidino-2-phenylindole (DAPI) to visualize nuclei (Sigma-Aldrich). Sections were examined for epifluorescence using a Zeiss Axioplan-2 fluorescence microscope (Carl Zeiss) equipped with the Axiovision program (version 4.7; Carl Zeiss).

### 2.6. Assessment of HDAC6 Activity 

HDAC6 activity was assessed in both rat and human cell samples using a commercially available fluorimetric assay kit (Biovision, Milpitas, CA, USA), employing a synthetic acetylated-peptide substrate resulting in the release of an AFC fluorophore, which can be detected and quantified with Ex/Em, 380/490 nm at 37 °C. 

### 2.7. Assessment of Thioredoxin-1 Activity 

Thioredoxin activity fluorescent assay kit (Cayman Chemical, Ann Arbor, MI, USA) was used to assess the activity of thioredoxin-1 (Trx-1) in rat retinal extracts and HuREC lysates. This assay measures the ability of endogenous Trx-1 to reduce the disulfides of fluorescently labeled insulin. The resulting fluorescent signal, measured at Ex/Em, 520/545 nm, is a direct measurement of Trx-1 reducing activity.

### 2.8. Protein Analysis 

Retinal tissue was homogenized in lysis buffer (ThermoFisher, Waltham, MA, USA) containing 1% phosphatase and protease inhibitor cocktail (Sigma-Aldrich). Protein concentration was measured using the Bio-Rad protein assay kit (Bio-Rad, Hercules, CA, USA) according to the manufacturer’s recommendation. Proteins from whole rat retinal tissue and HuREC lysates were separated by sodium dodecyl sulfate-polyacrylamide gel electrophoresis (SDS-PAGE) and transferred onto polyvinylidene difluoride (PVDF) membrane. The membrane was blocked using 5% skim milk and incubated with the following primary antibodies: anti-HDAC6 (Abcam, Cambridge, MA, USA), anti-Trx-1, anti-sirtuin 1 (SIRT1) and anti-albumin (all from Cell Signaling Technology). After incubation with horseradish peroxidase–conjugated secondary antibody (GE Healthcare, Pittsburg, PA, USA), bands were detected using the enzymatic chemiluminescence reagent, ECL (GE Healthcare). Subsequently, the membranes were stripped using stripping buffer (Bio-Rad) and re-probed with anti-β-actin antibody (Sigma-Aldrich) to assess equal loading. Scanned images of blots were used to quantify protein expression using NIH ImageJ software (http://rsb.info.nih.gov/ij/).

### 2.9. Dot BlotAanalysis 

Equivalent amount of proteins prepared from whole rat retinas and HuREC lysates were spotted on nitrocellulose membranes and dried for 5 min at room temperature. The membranes were blocked for 1 h by using 5 % non-fat dry milk in PBS and then probed for 1 h with either anti-3-nitrotyrosine (NT, Cayman) or anti 4-hydroxynonenal (4-HNE, Abcam) antibodies in PBS-tween buffer. The membranes were then washed three times in PBS-tween buffer and probed again with horseradish peroxidase-conjugate secondary antibody (Cell Signaling). After washing the membrane, the immuno-positive spots were visualized by using Clarity ECL- Blotting substrate (Bio-Rad). Scanned images of blots were used to quantify protein expression using NIH ImageJ software (http://rsb.info.nih.gov/ij/).

### 2.10. Quantitative PCR Analysis

Gene expression at mRNA level was assessed in retinal and HuREC extracts by quantitative polymerase chain reaction (qPCR). Total RNA was isolated from the HuREC and rat retinas using RNeasy Kit (Qiagen, Germantown, MD, USA). cDNA was prepared using iScript™ cDNA Synthesis Kit (Bio-Rad). Amplification of HDAC6, Trx-1, GCLC, GCLM, NQO1, and HO-1 mRNA was performed using power SYBR green PCR master mix (Applied Biosystems, Foster City, CA, USA). The conditions used for the PCR were as it follows: 95 °C for 3 min (1 cycle) and 94 °C for 20 s, 55 °C for 30 s, and 72 °C for 40 s (40 cycles). The relative mRNA abundance was determined by normalizing to mRNA for hypoxanthine phosphoribosyltransferase 1 (HPRT-1) for tissue or 18 s for cells, using the 2Ct method (Ct refers to the threshold value). A complete list of the different primers used in this study is included in Supplementary Table S3.

### 2.11. Reactive Oxygen Species Assays 

For detection of superoxide in retinal tissue, 10 μm-thick retinal cryosections, from different experimental groups, were covered (at room temperature) with a 10 μM dihydroethidium (DHE) solution and incubated in a light-protected humidified incubator at 37 °C for 30 min. At the end of the incubation, sections were mounted with a coverslip and images were taken using Zeiss Axioplan-2 fluorescence microscope (Carl Zeiss). The fluorescence intensity was measured using NIH ImageJ software (http://rsb.info.nih.gov/ij/). 

ROS detection from cellular sources was accomplished by CellROX green assay (ThermoFisher) performed according to the manufacturer’s protocol. HuREC were loaded with 5 µM CellROX green in culture medium and stained in the dark for 30 min at 37 °C. Stained cells were washed in PBS twice, mounted using Fluoroshield mounting medium containing DAPI (Sigma-Aldrich) to visualize nuclei. Images were then immediately captured using a Zeiss Axioplan-2 fluorescence microscope (Carl Zeiss). 

Mitochondrial superoxide production was measured using MitoSOX Red (ThermoFisher). HuREC were loaded with 5 µM MitoSOX red in Hank’s balanced salt solution (HBSS) with calcium and magnesium for 30 min at 37 °C in the dark. Stained cells were then washed and suspended in HBSS, mounted using Fluoroshield mounting medium containing DAPI (Sigma-Aldrich) and immediately analyzed under a Zeiss Axioplan-2 fluorescence microscope (Carl Zeiss).

### 2.12. Assessment of Senescence Markers

To evidence senescent HuREC, we used senescence-associated β-Galactosidase (SA-β-Gal) reactivity-based assay using a commercially available kit (Cell Signaling) as previously shown [7]. Positive reactivity to SA-β-Gal, assessed at pH 6, is measured on images captured (10 frames per well) at 20× magnification by light microscopy using Zeiss Axioplan-2 microscope (Carl Zeiss). Percentage of SA-β-Gal positive cells/well was determined as number of cells positive for a blue color versus total number of cells counted in a blind fashion. 

### 2.13. Statistical Analysis

Graphs were prepared using Graph Pad Prism 8.0 software for Windows (Graph Pad Software, San Diego, CA, USA). Data are shown as means ± standard error of mean (SEM). Statistical significance among experimental groups was established using one-way ANOVA, followed by the Bonferroni multiple-comparison test. Differences were considered significant when *p* was <0.05.

## 3. Results

### 3.1. HDAC6 Expression and Activity are Increased in the Diabetic Retina

**Figure 1:**
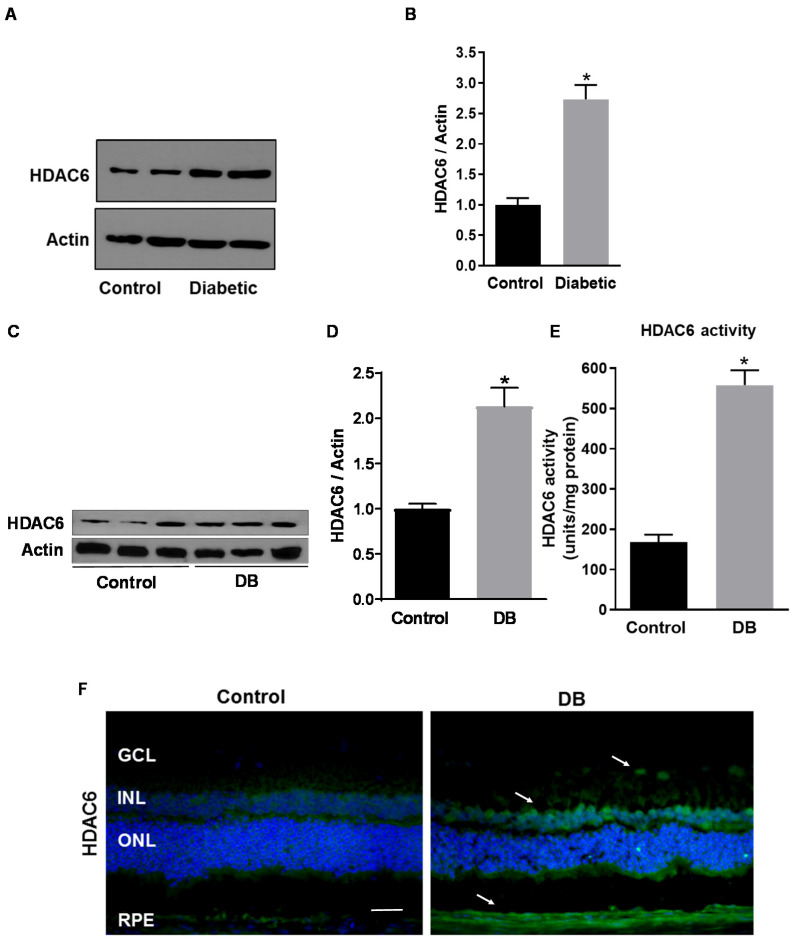
Histone deacetylase 6 (HDAC6) expression in the diabetic retina. (**A**) Western blotting analysis measuring HDAC6 protein levels in human postmortem retinas from diabetic and non-diabetic donors (control). (**B**) Bar histograms representing relative optical densities from the immunoblotting shown in (A) and normalized versus the loading control actin. Values are expressed as mean ± SEM for *n* = 8. * *p* < 0.01 vs. control. (**C**) Western analysis of HDAC6 protein expression in retinas of streptozotocin-induced diabetic rats (STZ-rats) (DB) at 8 weeks of hyperglycemia and age-matched normoglycemic control rats (control). (**D**) Bar histograms representing densitometric quantification of HDAC6 protein levels normalized to actin. (**E**) HDAC6 activity measured, by a fluorimetric assay, in retinas of STZ-rats and control normoglycemic rats. (**F**) Representative microimages of immunohistochemical analysis of HDAC6 (green) in retinas of STZ-rats at 8 weeks of hyperglycemia and of age-matched normoglycemic control rats. Nuclei were stained with 4′,6-diamidino-2-phenylindole (DAPI). White arrows indicate areas of increased immunoreactivity. Scale bar, 50 µm. Values are expressed as mean ± SEM for *n* = 6. * *p* < 0.01 vs. control.

HDAC6 expression and activity were measured in human DR using postmortem human retinas from diabetic and non-diabetic donors and STZ-rats compared to normoglycemic age-matched control. As shown in Figure 1A,B, Western blotting analysis showed a 2.5-fold increase in HDAC6 expression in retinas of postmortem diabetic donors as compared to retinas of non-diabetic donors (*p* < 0.003; *n* = 8). We then measured the expression and retinal tissue distribution of HDAC6 in STZ-rats (8 weeks of hyperglycemia) compared to normoglycemic age-matched control rats. Western blotting analysis (Figure 1C,D) showed a 2.2-fold increase of HDAC6 protein levels in retinas of STZ-rats at 8 weeks of hyperglycemia in comparison to age-matched normoglycemic control rats (*p* < 0.006; *n* = 6). Further, HDAC6 enzymatic activity, measured with a fluorimetric assay, was significantly increased in retinas of STZ-rats when compared to normoglycemic age-matched control rats (*p* < 0.001; *n* = 6) (Figure 1E). Finally, immunohistochemical analysis of normal and diabetic rat retinal sections (Figure 1F), confirmed HDAC6 increased expression in diabetic rat retinas and showed its immunolocalization in several retinal layers, particularly in the inner nuclear layer (INL), retinal pigmented epithelium (RPE), and around retinal blood vessels in the ganglion cell layer (GCL) (white arrows in Figure 1F).

### 3.2. Tubastatin A Decreases the Expression and Activity of HDAC6 in the Diabetic Retina

**Figure 2:**
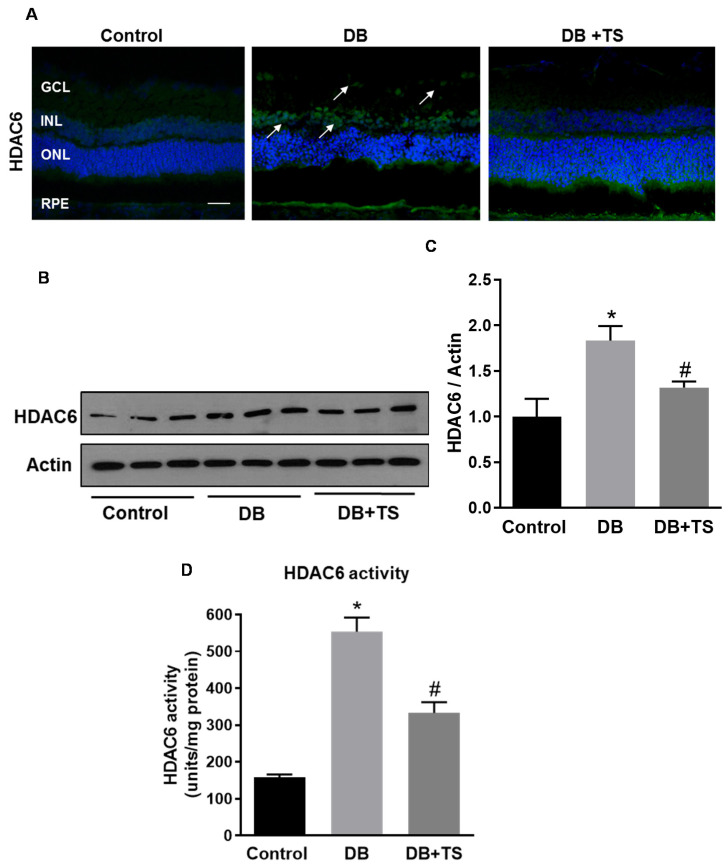
Effects of Tubastatin A on HDAC6 expression and activity. (**A**) Representative images of immunohistochemical analysis of HDAC6 (green) of retinal cryosections of STZ-rats (DB) (8 weeks of hyperglycemia), age-matched normoglycemic control rats, and STZ-rats receiving TS 10 mg/kg (DB + tubastatin A (TS)). Nuclei were stained with DAPI. Scale bar, 50 µm. (**B**) Western analysis assessing HDAC6 protein levels in STZ-rats after 8 weeks of hyperglycemia (DB), STZ-rats treated with 10 mg/kg TS (DB +TS) and age-matched normoglycemic rats (control). (**C**) Bar histograms representing densitometric values of HDAC6 protein expression measured in the different experimental groups and normalized to actin. (**D**) HDAC6 activity measured, by fluorimetric assay, in the three experimental groups (control, DB and DB + TS). Values are mean ± SEM for *n* = 6. * *p* < 0.05 vs. control and ^#^ *p* < 0.05 vs. DB.

Next, we determined the effect of the HDAC6 specific inhibitor Tubastatin A (TS), on diabetes-induced increase in HDAC6 expression and activity in the retina of diabetic rats. STZ-rats were treated with 10 mg/kg of TS, administered intraperitoneally every other day starting two weeks after the onset of diabetes and prolonged for another 6 weeks (total 8 weeks of diabetes). As shown in Figure 2A, TS treatment resulted in a marked reduction of HDAC6-specific immunoreactivity in comparison to untreated STZ-rats (DB). Western blotting analysis confirmed these data by showing a significant reduction in HDAC6 protein levels in retinas of TS-treated STZ-rats (DB + TS) in comparison with untreated STZ-diabetic rats (DB) (*p* < 0.05; *n* = 6) (Figure 2B,C). As expected, we also observed a significant decrease in HDAC6 enzymatic activity (Figure 2D) in retinas of TS-treated STZ-rats (DB + TS) in comparison to untreated STZ-rats (DB) (*p* < 0.01; *n* = 6). 

### 3.3. Tubastatin A Preserves Retinal Structural Morphology and Reduces Vascular Leakage in Diabetic Retina

**Figure 3:**
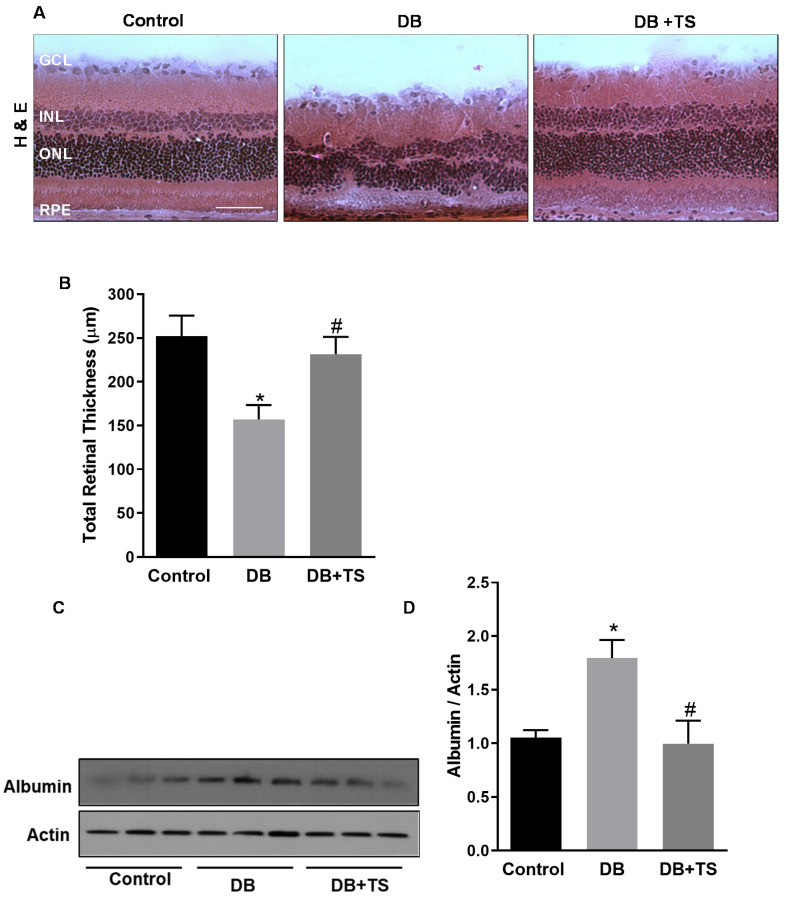
Effects of Tubastatin A on retinal histopathology and vascular leakage. (**A**) Hematoxylin and eosin (H&E) staining of retinal cryosections assessing retinal morphology of STZ-rats (DB), STZ-rats receiving TS (10 mg/kg) (DB + TS) and normoglycemic control rats (control). Scale bar, 50 µm. (**B**) The bars represent retinal thickness values measured in H&E retinal cryosections obtained from the different treatment groups. (**C**) Western analysis of albumin extravasation in retinas of diabetic STZ-rats (DB), STZ-rats receiving 10 mg/kg TS (DB + TS) and normoglycemic control rats (control). (**D**) Bar histograms representing optical density of albumin normalized to actin. Values are mean ± SEM for *n* = 6. * *p* < 0.05 vs. control and ^#^ *p* < 0.05 vs. DB.

Morphological and morphometric analyses were conducted evaluating retinal cryosections stained with hematoxylin and eosin to assess the effects of TS treatment on retinal histopathology (Figure 3A,B). Figure 3B shows that total retinal thickness was significantly reduced in diabetic rats after 8 weeks of hyperglycemia (DB) in comparison to control age-matched normoglycemic rats (control) (*p* < 0.03). Treatment of diabetic rats with TS (DB + TS) normalized the morphology of the retinal layers as shown by a significant preservation of total retinal thickness (Figure 3B) (*p* < 0.05) compared to untreated diabetic rats (DB).

Blood–retinal barrier (BRB) dysfunction, measured as an increase in vascular permeability, is an important evidence of diabetes-induced retinal vascular abnormalities [19,20]. To determine the effect of TS on hyperglycemia-induced vascular leakage in the diabetic retina, we assessed albumin extravasation after perfusion in retinas of control, DB and DB + TS rats by Western blotting. As shown in Figure 3C,D, extravascular albumin levels were significantly higher in retinas of STZ rats (DB) when compared to normoglycemic age-matched rats (control), whereas treatments with TS (DB + TS) significantly reduced albumin leakage in diabetic rats (*p* < 0.02 vs. control and *p* < 0.05 vs. DB; *n* = 6).

### 3.4. Tubastatin A Decreases the Levels of Senescence Markers in the Diabetic Retina 

We previously showed that diabetes promotes retinal vascular senescence and this effect is associated with loss of the NAD + -dependent histone deacetylase sirtuin 1 (SIRT1) and up-regulation of senescence markers [4,7]. We, therefore, determined whether inhibition of HDAC6 by TS affected this mechanism in the diabetic retina. 

**Figure 4:**
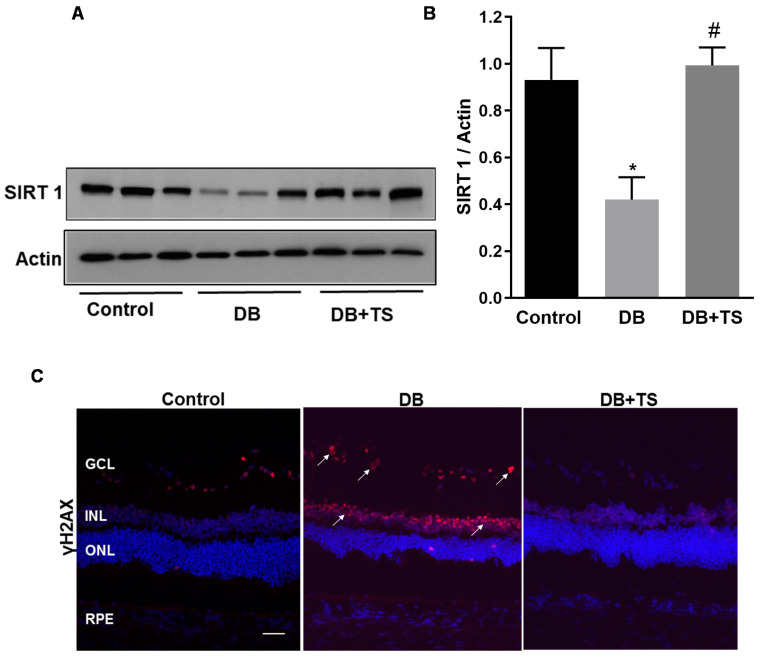
Effects of Tubastatin A on senescence in diabetic retina. (**A**) Immunoblot showing protein expression levels of SIRT1 measured in retinal extracts of STZ-rats (DB), STZ-rats receiving TS (10 mg/kg) (DB + TS) and age-matched normoglycemic control rats. (**B**) Bar histograms representing densitometric quantification of SIRT1 immunoblotting normalized to the loading control actin. (**C**) Representative images of immunohistochemical analysis of γH2AX (red) in retinas of STZ-rats (DB), STZ-rats treated with TS (DB + TS) and age-matched normoglycemic control rats (control). Nuclei were stained with DAPI. Scale bar, 50 µm. Values are mean ± SEM for *n* = 6. * *p* < 0.05 vs. control and ^#^ *p* < 0.01 vs. DB.

Expression of SIRT1 was analyzed by Western blotting in retinas of rats from the different experimental groups (control, DB and DB + TS rats). As shown in Figure 4A,B, we found that SIRT1 expression was significantly decreased in the diabetic group (DB) compared to normoglycemic control and treatments of the diabetic rats with TS partially rescued it (*p* < 0.05 vs. control and *p* < 0.01 vs. DB; *n* = 6). Furthermore, immunohistochemical analysis of the senescence marker the phosphorylated form of H2A histone family member X (γH2AX) showed (Figure 4C) increased immunoreactivity in the diabetic rat retinas as compared to control group, particularly in the INL and in GCL (white arrows, Figure 4C). However, in TS-treated diabetic retinas, γH2AX-specific immunofluorescence was markedly decreased compared to STZ-rat retinas (Figure 4C). 

### 3.5. Tubastatin A Decreases Hyperglycemia-Induced Oxidative/Nitrative Stress in Retina 

**Figure 5:**
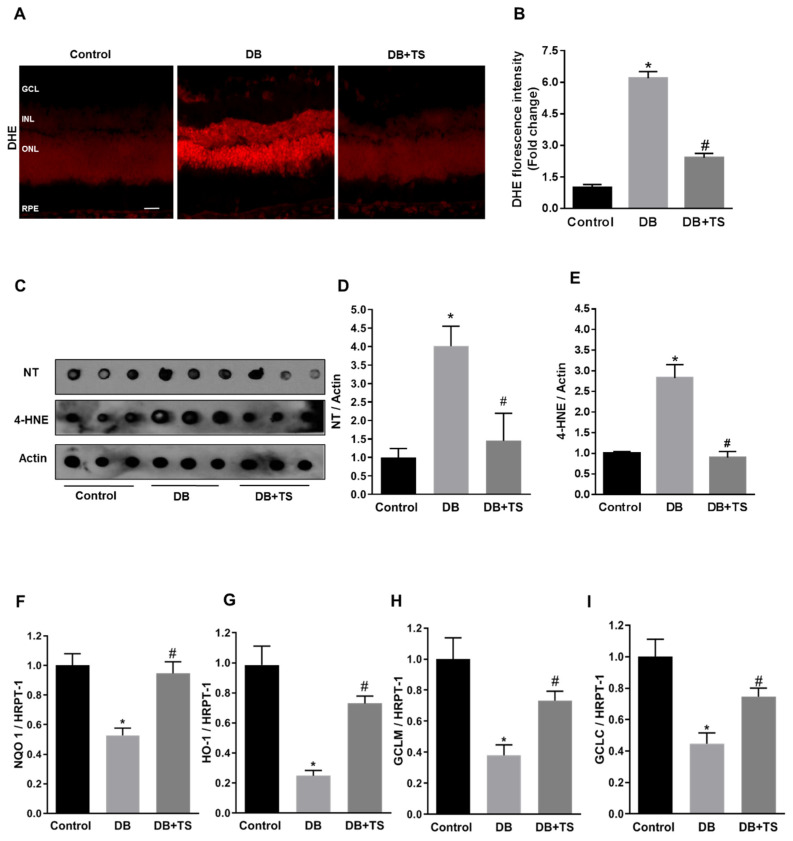
Effects of Tubastatin A on retinal redox homeostasis. (**A**) Representative images of retinal cryosections from the different experimental groups (control, DB and DB + TS) probed with dihydroethidium (DHE) to detect superoxide. Scale bar, 50 μm. (**B**) Quantification of relative fluorescence intensity of DHE staining. Values are mean ± SEM for *n* = 6. Results are presented as a fold change of control. * *p* < 0.01 vs. control and ^#^ *p* < 0.01 vs. DB. (**C**) Dot blot analysis assessing levels of nitrotyrosine (NT) and 4-hydroxynonenal (4-HNE) in retinas of three experimental groups (control, DB and DB + TS rats). (**D**,**E**) Quantification of optical density of NT and 4-HNE immunoblotting normalized versus actin. Values are mean ± SEM for *n* = 6. * *p* < 0.05 vs. control and ^#^ *p* < 0.05 vs. DB for NT. * *p* < 0.01 vs. control and ^#^ *p* < 0.01 vs. DB for 4-HNE. (**F**–**I**) mRNA levels of heme oxygenase-1 (HO-1), NAD(*p*)H dehydrogenase quinone 1 (NQO1), glutamate-cysteine ligase regulatory subunit (GCLM) and glutamate-cysteine ligase (GCLC) evaluated by qPCR and normalized to mRNA for hypoxanthine phosphoribosyltransferase 1 (HPRT-1). Values are mean ± SEM for *n* = 6. * *p* < 0.05 vs. control and ^#^ *p* < 0.05 vs. DB.

Increased oxidative/nitrative stress has been shown to be a key pathogenic hub for the development of DR [4,21]. To understand the potential role of HDAC6 in this process, we investigated TS effects on hyperglycemia-induced oxidative/nitrative stress by measuring retinal levels of superoxide, by dihydroethidium (DHE) staining and nitrotyrosine (NT) and 4-hydroxynonenal (4-HNE) by dot-blot analysis. Retinal cryosections probed with DHE fluorescent staining, showed increased fluorescence intensity in the diabetic rat retinas (DB) compared to normoglycemic group (control) (Figure 5A). This effect was markedly reduced by treatment of the diabetic rats with TS (Figure 5A). Quantification of fluorescence intensity confirmed our staining data (*p* < 0.01 vs. control and *p* < 0.01 vs. DB; *n* = 6) (Figure 5B). Accordingly, dot blot analysis of retinal levels of NT and 4-HNE showed that TS treatment prevented the increase of both these markers that we observed in diabetic rats (*p* < 0.05 vs. control and *p* < 0.05 vs. DB for NT and *p* < 0.01 vs. control and *p* < 0.01 vs. DB for 4-HNE; *n* = 6) (Figure 5C–E).

### 3.6. Tubastatin A Restores Antioxidant Activity in the Diabetic Retina

Redox stress in the diabetic retina could result from increased oxidase activities, but also from reduced endogenous antioxidant activities. Nuclear factor erythroid-2-related factor 2 (Nrf2) is a master regulator of endogenous antioxidants gene expression [22], therefore, we tested the effect of TS on the regulation of Nrf2-dependent antioxidant signaling, by monitoring, the expression levels of well-established Nrf2–dependent gene targets. QPCR analysis revealed that diabetes promoted a significant reduction in the expression levels of the Nrf2-dependent genes: Heme oxygenase-1 (HO-1), NAD(*p*)H dehydrogenase quinone 1 (NQO1), glutamate-cysteine ligase regulatory subunit (GCLM), and glutamate-cysteine ligase (GCLC) (Figure 5F–I) However, treatment of diabetic rats with TS restored the mRNA levels of all these genes (*p* < 0.005 (HO-1), *p* < 0.01 (NQO1), *p* < 0.02 (GCLM), *p* < 0.02 (GCLC) vs. control and *p* < 0.002 (HO-1), *p* < 0.01 (NQO1), *p* < 0.02 (GCLM), *p* < 0.03 (GCLC) vs. DB; *n* = 6), thus, suggesting that TS restored Nrf2-dependent signaling in the diabetic retina. 

**Figure 6:**
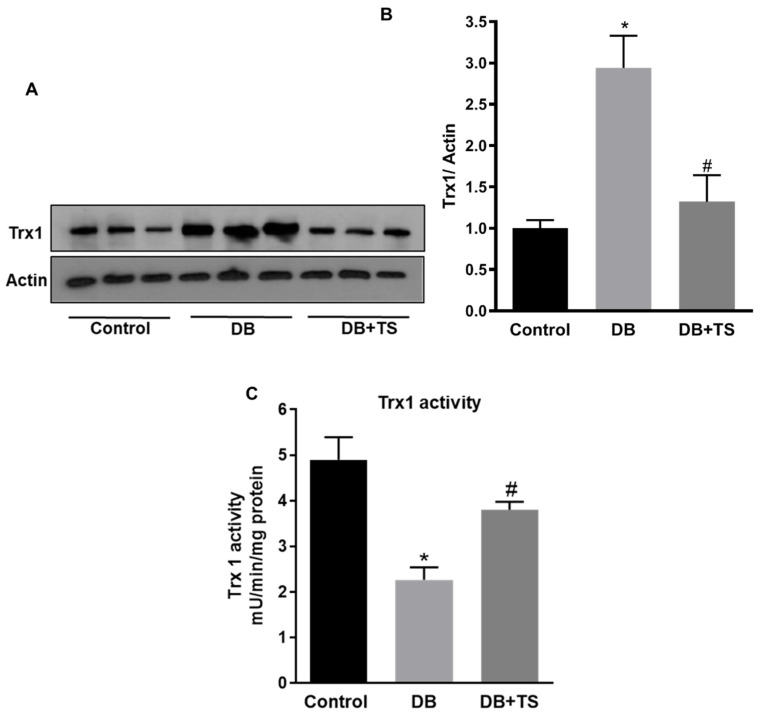
Effect of Tubastatin A on thioredoxin-1 expression and activity. (**A**) Western analysis of thioredoxin-1 (Trx-1) protein expression measured in retinas of STZ-rats (DB), STZ-rats receiving TS (10 mg/kg) (DB + TS) and normoglycemic control rats (control). (**B**) Quantification of optical density of Trx-1 immunoblotting normalized versus actin. (**C**) Trx-1 enzymatic activity measured in STZ-rats (DB), STZ-rats treated with TS (10 mg/kg) (DB + TS). Values are mean ± SEM for *n* = 6. * *p* < 0.01 vs. control and # *p* < 0.01 vs. DB.

Furthermore, we assessed the expression and activity of the endogenous antioxidant Trx-1 (Figure 6A,B). As previously reported [8], Trx-1 expression was significantly increased in retinas of STZ-rats (DB) in comparison to normoglycemic control rats (Figure 6A,B). Treatment of the diabetic rats with TS, however, significantly decreased Trx-1 expression in diabetic rats (*p* < 0.0051; *n* = 6). Trx-1 activity, measured with a fluorimetric assay, was found to be significantly lower in retinas of STZ-rats (DB) than in normoglycemic age-matched rats (control) (Figure 6C). However, treatment of diabetic rats with TS rescued/normalized this antioxidant enzymatic activity (*p* < 0.01 vs. DB; *n* = 6) (Figure 6C).

### 3.7. Effect of High Glucose and Tubastatin A on HDAC6 Expression and Activity in Human Retinal Endothelial Cells 

**Figure 7:**
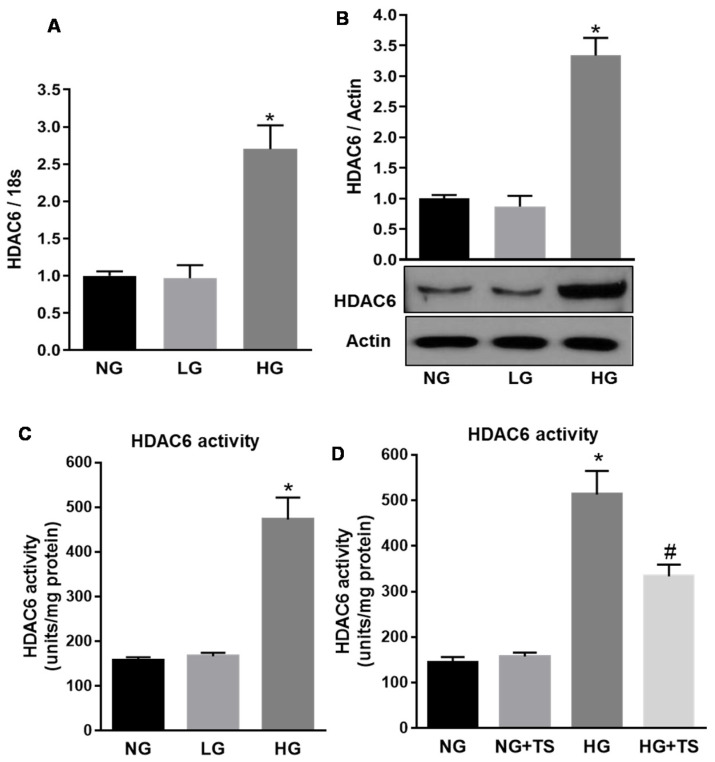
Effects of high glucose and Tubastatin A on HDAC6 expression and activity in HuREC. (**A**) HDAC6 mRNA expression, measured by qPCR, in HuREC exposed to different glucose levels (NG = 5.5 mM d-glucose, HG = 25 mM d-glucose) and the osmotic control l-glucose (25 mM) for 48 and 72 h. (**B**) Immunoblotting showing HDAC6 protein levels measured 48 h after exposure of HuREC to HG or the controls NG or LG. (**C**) HDAC6 activity measured in HuREC by fluorimetric assay at 48 h exposure of the cells to HG, NG or LG. (**D**) HDAC6 activity measured in HuREC by fluorimetric assay after 48 h of exposure of the cells to HG or HG plus TS (5 µM starting 6 h before HG treatment) and compared to the controls NG or LG. Values are mean ± SEM for *n* = 3. * *p* < 0.01 vs. NG and ^#^ *p* < 0.01 vs. HG.

To determine the specific impact of HDAC6 on retinal endothelial cells and microvascular dysfunction, we performed experiments in vitro using HuREC exposed to different glucose levels. First, we confirmed that HDAC6 mRNA expression levels, measured in HuREC by qPCR analysis, were significantly increased when the cells were treated with high glucose concentrations (HG, 25 mM) as compared to cells treated with the osmotic control l-glucose (LG) or exposed to normal glucose containing media (NG, 5.5 mM) (*p* < 0.01 vs. NG; *n* = 3) (Figure 7A). Accordingly, HDAC6 protein expression (Figure 7B) was also significantly augmented in HG-treated HuREC in comparison with LG or NG (*p* < 0.01; *n* = 3). Parallel to HDAC6 protein up-regulation, we also found that HG increased HDAC6 activity 48 h post-treatment in comparison to NG and LG controls (Figure 7C) (*p* < 0.005; *n* = 3). Moreover, treatment of HuREC with TS (5 μM, 6 h pre-treatment + 48 h in combination with HG) significantly down-regulated the activity of HDAC6 in HuREC exposed to HG (*p* < 0.01; *n* = 3) and had no significant effects on cells exposed to normal glucose control (NG) (Figure 7D). 

### 3.8. Effects of HDAC6 Inhibition on Oxidative/Nitrative Stress and Endogenous Antioxidants in HuREC

**Figure 8:**
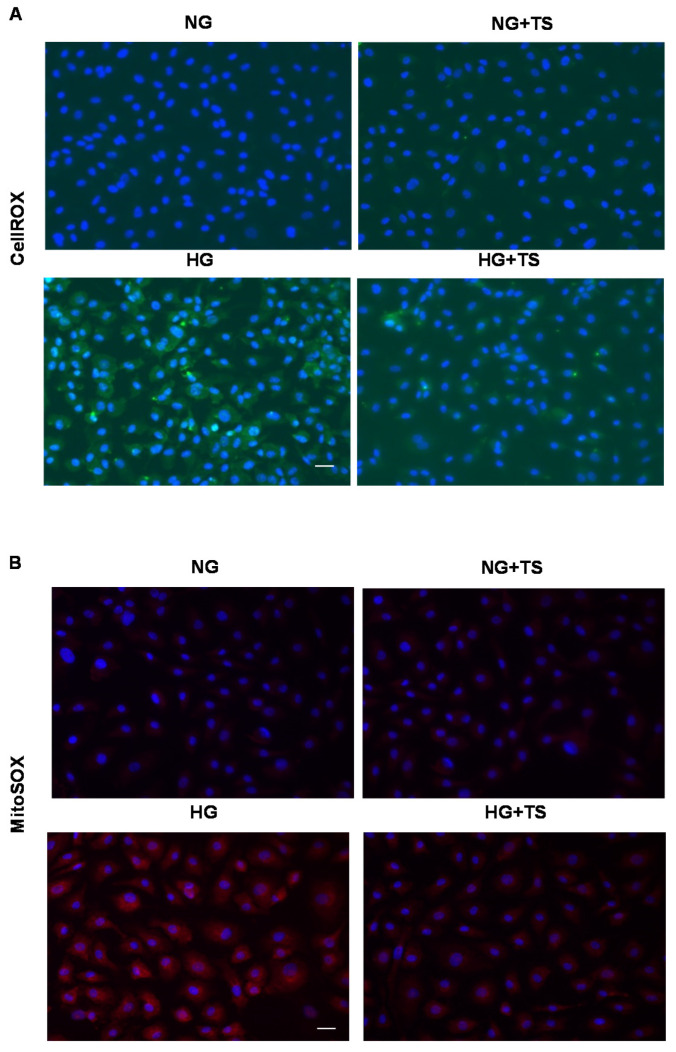
Effects of Tubastatin A on cellular and mitochondrial oxidases activities in HuREC. (**A**) CellROX fluorescent assay showing superoxide formation (green) in HuREC exposed to HG for 48 h or to HG in the presence of 5 µM of TS (HG + TS) and compared to HuREC cultured in NG conditions in the absence (NG) or in presence of 5 µM TS (NG + TS). (**B**) Images of MitoSOX assay showing superoxide formation from mitochondria oxidase (red) in HuREC exposed to HG for 48 h or HG in the presence of 5 µM of TS (HG + TS), also for 48 h, and compared to HuREC cultured in NG conditions in the absence (NG) or in the presence of 5 µM TS (NG + TS). In A and B blue fluorescence show cell nuclei counterstained with DAPI. Scale bar, 50 µm.

To explore the potential contribution of HDAC6 to HG-induced redox imbalance in HuREC, we assessed the formation of ROS from cellular sources by determining CellROX deep green fluorescence intensity in HuREC exposed to NG or HG for 48 h with or without TS (5 μM) (Figure 8A). We found that HG increased superoxide-dependent fluorescence intensity in HuREC as compared to NG group, however, this effect was largely blocked by TS (Figure 8A). 

Same results were obtained while monitoring the effects of HG and TS on superoxide production from mitochondrial sources (Figure 8B). Analysis of mitochondrial oxidases activity by MitoSOX, showed that exposure of HuREC to HG for 48 h increased mitochondrial superoxide-dependent reactivity; however, TS prevented this effect (Figure 8B). In all cases, TS treatment did not affect the response of the cells to NG (NG + TS). 

**Figure 9:**
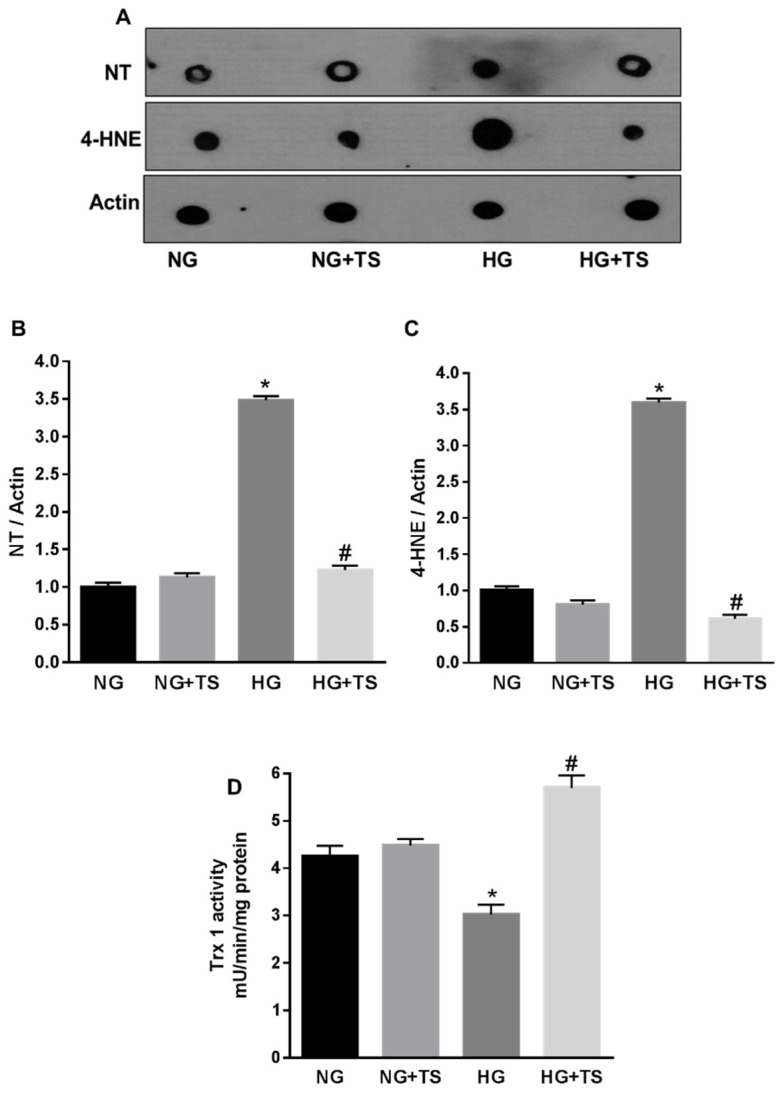
Effects of Tubastatin A on high glucose-induced redox imbalance in HuREC. (**A**) Representative images of dot blot analysis demonstrating nitrotyrosine (NT) and 4-hydroxynonenal (4-HNE) formation in HuREC exposed to HG for 48 h or HG in the presence of 5 µM of TS (HG + TS), also for 48 h, and compared to HuREC cultured in NG conditions in the absence (NG) or presence of 5 µM TS (NG + TS). (**B**,**C**) Quantification of optical density of NT and 4-HNE immunoblotting normalized versus actin. Values are mean ± SEM for *n* = 3. * *p* < 0.0001 vs. NG and # *p* < 0.0001 vs. HG. (**D**) Fluorimetric assay results representing Trx-1 activity in HuREC assessed after 48 h of exposure to different glucose levels (NG = 5.5 mM d-glucose, HG = 25 mM d-glucose) in the presence or absence of TS (5 μM). Values are mean ± SEM for *n* = 3. * *p* < 0.01 vs. NG and ^#^ *p* < 0.01 vs. HG.

Furthermore, dot blot analysis showed that the levels of the oxidative/nitrative stress markers NT and 4-HNE, were increased by HG, however treatment of the cells with TS halted this effect of HG (*p* < 0.0001 vs. NG and *p* < 0.0001 vs. HG; *n* = 3) (Figure 9A–C). To ascertain whether TS was also able to normalize endogenous antioxidants, we determined the effects of HG in presence and/or absence of TS on Trx-1 activity (Figure 9D). Similarly, to what was observed in the diabetic rats, glucidic stress (HG) significantly decreased Trx-1 activity in HuREC and this was rescued by TS (*p* < 0.001 vs. NG and *p* < 0.01 vs. HG; *n* = 3) (Figure 9D).

### 3.9. Effects of HDAC6 Inhibition on HG-induced HuREC Senescence 

**Figure 10:**
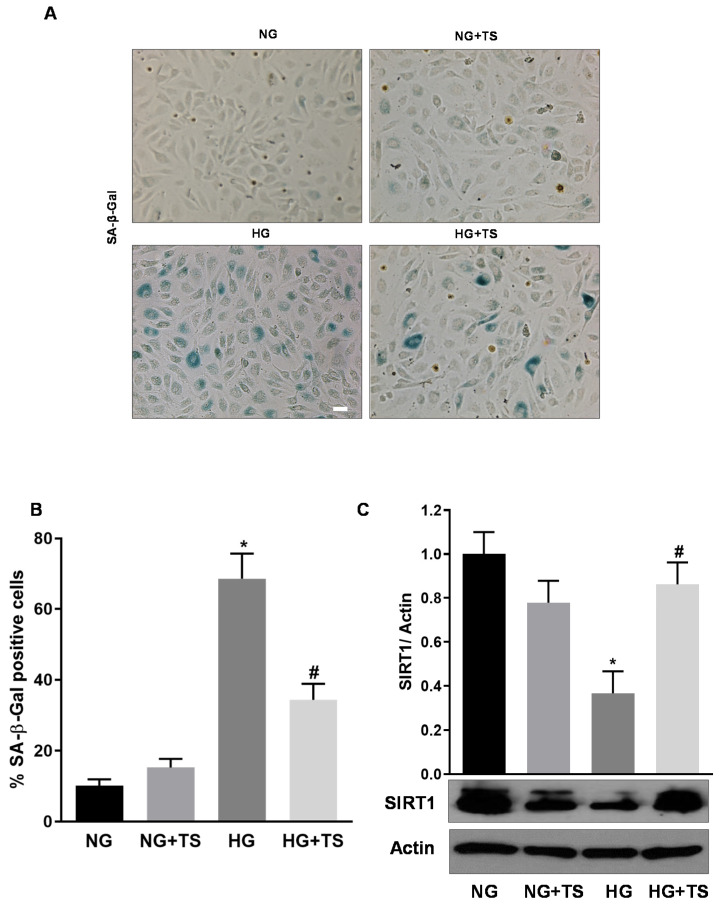
Tubastatin A effects on HG-induced senescence markers in HuREC. (**A**) Representative images of senescence-associated β-Galactosidase (SA-β-Gal) reactivity assay in HuREC exposed to HG for 48 h or HG plus 5 µM of TS (HG + TS), also for 48 h, and compared to HuREC cultured in NG conditions in the absence (NG) or presence of 5 µM TS (NG + TS). Positive cells develop the blue color. Scale bar, 50 µm. (**B**) Quantification of SA-β-Gal-positive cells. Values are number of positive cells per well versus total number of cells expressed as a percent. *n* = 3. * *p* < 0.001 vs. NG and ^#^ *p* < 0.001 vs. HG. (**C**) Western blotting analysis showing protein levels of the histone deacetylase SIRT1 in HuREC treated in the same experimental conditions as described in (**A**). Bar histograms represent optical density values of the blots normalized for the loading control actin. Values are mean ± SEM for *n* = 3. * *p* < 0.01 vs. NG and ^#^ *p* < 0.01 vs. HG.

Finally, we examined the effects of HDAC6 inhibition by TS on HG-induced HuREC senescence. Assessment of SA-β-Gal activity in HuREC exposed to different glucose conditions showed increased number of positive cells in the HG treatment group compared to the NG control (*p* < 0.001; *n* = 3) (Figure 10A,B). However, treatment of HuREC with TS prevented the increase of SA-β-Gal–reactive cells in HG conditions (*p* < 0.005; *n* = 3) (Figure 10A,B). 

Moreover, Western analysis of protein levels of the histone deacetylase SIRT1 showed that this was significantly down-regulated in HG-treated HuREC in comparison to NG. Treatment of the cells with TS significantly reduced the effects of HG by rescuing SIRT1 protein levels (*p* < 0.005 vs. HG; *n* = 3) (Figure 10C).

## 4. Discussion

The increased incidence of diabetes and DR, urges the realization of early interventional therapeutic strategies. In this study, we investigated the role of the histone deacetylase HDAC6 in the early events characterizing the progression of an experimental model of DR. Dysregulation of the processes of acetylation/deacetylation of nuclear and cellular proteins has been shown to be associated with different pathologic conditions including diabetes [12,13,14,15,16,17,18,23]. Previous findings linked the activity of several histone deacetylases to the pathogenesis of DR [23,24]. A specific contribution of HDAC1, 2, and 8 to global acetylation of retinal histones in the diabetic retina was found to be involved not only to progression of DR but also to the metabolic memory phenomenon [23]. When compared to the other members of the large family of the histone deacetylases HDAC6 presents unique structural and functional properties, including a double catalytic domain and cytosolic and nuclear intracellular localization and sites of action [9]. Among other unique functions, HDAC6 can influence the redox state of the cells through deacetylation of endogenous antioxidants and modulation of oxidase activities [16]. 

Our results show that HDAC6 expression and activity are upregulated in the diabetic retinas of STZ-rats and, most importantly, in postmortem retinas of diabetic donors. Using an interventional approach, we assessed the effects of pharmacological inhibition of HDAC6 by administration of the specific inhibitor TS in an experimental model of Type 1 diabetes (STZ-rats). Pharmacological inhibition of HDAC6 by TS lessened DR pathology in STZ-rats, as evidenced by maintenance of total retinal thickness and amelioration of vascular hyperpermeability, a key feature of DR microangiopathy [2,3]. The observed effects of TS on BRB stabilization in STZ-rats, are in agreement with previous studies that have underscored the role of HDAC6 activity in altering endothelial and epithelial cells permeability and the stability of intercellular junctions [25,26,27]. 

While the specific role of HDAC6 in the diabetic retina has not been studied before, HDAC6 inhibition has been shown to alleviate myocardial ischemia-reperfusion injury in diabetic rats [16] and to ameliorate diabetic kidney disease [15], predominantly through antioxidant effects. Interestingly, in the diabetic retina, downregulation of HDAC6 was linked to the effects of exogenous GLP-1 in alleviating oxidative stress-induced apoptosis and autophagy of retinal cells [24].

The results of our studies show that HDAC6 inhibition by TS decreased the appearance of the senescence marker γH2AX and significantly augmented the expression of the redox homeostatic histone deacetylase SIRT1, of which expression levels are inversely correlated with the induction of endothelial cell senescence [4,7]. A contrasting balance between HDAC6 and SIRT1 could significantly impact the redox status of a number of cells including endothelial and other retinal cells [28]. 

Previous studies have shown that increased HDAC6 activity results in oxidative stress due to mitochondrial dysfunction [16,29] and to altered endogenous antioxidant function [11]. Increased oxidative/nitrative stress has been shown to play a key contributing role to the pathogenesis of DR due to its effects in promoting retinal chronic inflammation, microvascular injury, and accelerated endothelial cell senescence [4]. Inhibition of HDAC6 by TS, significantly reduced superoxide formation, diminished the levels of oxidative and nitrative stress markers (4-HNE and NT, respectively) and rescued the activity of endogenous antioxidants such as Nrf2 and Trx-1. Taken together, these antioxidant properties of TS further confirm the pro-oxidative capacity of overactive HDAC6 in the diabetic retina. 

The results of our experiments in vitro, specifically addressing HDCA6 role in HuREC, largely recapitulated the results obtained in the in vivo experiments as TS prevented high glucose-induced HuREC senescence (SA-β-Gal activity assay) and halted high glucose effects in downregulating SIRT1 expression. These protective effects correlated with significant reduction of superoxide production from cellular and mitochondrial sources as well as with the rescuing of the activity of the endogenous antioxidant Trx-1. Consistent with our results, blocking of HDAC6 activity was shown to be protective against high glucose-induced oxidative stress in RPE via mechanisms involving inhibition NF-κB and NLRP3 inflammasome pathway [30].

The potential role of HDAC6 in regulating cellular redox homeostasis has been previously implicated in the pathogenesis of several neurodegenerative diseases [31,32]. In addition, similar findings have been obtained in studies showing beneficial antioxidant effects of HDAC6 inhibitors in models of retinal neurodegenerative diseases [17,18]. Thus, the results here described are in agreement with these previous findings by demonstrating a role for HDAC6 in DR and retinal microangiopathy through pro-oxidant effects. 

## 5. Conclusions

In summary, the results of the studies described herein, are the first to demonstrate the impact of HDAC6 activation in the diabetic retina and to suggest the potential therapeutic efficacy of HDAC6 specific inhibitors for the prevention of redox imbalance and injury to the retinal microvasculature in the diabetic milieu.

## Supplementary Materials

The following are available online at https://www.mdpi.com/2076-3921/9/7/599/s1, Table S1: Demographics and clinical history of human postmortem retinal donors. Table S2: Biochemical parameters measured in the experimental rat groups. Table S3: Primer sequences used in the study. Figure S1: MTT Assay.

## References

[1] Ogurtsova K., da Rocha Fernandes J.D., Huang Y., Linnenkamp U., Guariguata L., Cho N.H., Cavan D., Shaw J.E., Makaroff L.E. (2017). IDF Diabetes Atlas: Global estimates for the prevalence of diabetes for 2015 and 2040. Diabetes Res.Clin. Pr..

[2] Antonetti D.A., Klein R., Gardner T.W. (2012). Diabetic retinopathy. New Engl. J. Med..

[3] Duh E.J., Sun J.K., Stitt A.W. (2017). Diabetic retinopathy: Current understanding, mechanisms, and treatment strategies. JCI Insight.

[4] Lamoke F., Shaw S., Yuan J., Ananth S., Duncan M., Martin P., Bartoli M. (2015). Increased oxidative and nitrative stress accelerates aging of the retinal vasculature in the diabetic retina. PLoS ONE.

[5] Kowluru R.A. (2019). Mitochondrial stability in diabetic retinopathy: Lessons learned from epigenetics. Diabetes.

[6] Kowluru R.A., Mishra M. (2015). Oxidative stress, mitochondrial damage and diabetic retinopathy. Biochim. Biophys. Acta.

[7] Thounaojam M.C., Jadeja R.N., Warren M., Powell F.L., Raju R., Gutsaeva D., Khurana S., Martin P.M., Bartoli M. (2019). MicroRNA-34a (miR-34a) mediates retinal endothelial cell premature senescence through mitochondrial dysfunction and loss of antioxidant activities. Antioxidants.

[8] Thounaojam M.C., Powell F.L., Patel S., Gutsaeva D.R., Tawfik A., Smith S.B., Nussbaum J., Block N.L., Martin P.M., Schally A.V. (2017). Protective effects of agonists of growth hormone-releasing hormone (GHRH) in early experimental diabetic retinopathy. Proc. Natl. Acad. Sci. USA.

[9] Liang T., Fang H. (2018). Structure, functions and selective inhibitors of HDAC6. Curr. Top. Med. Chem..

[10] Choi H., Kim H.J., Kim J., Kim S., Yang J., Lee W., Park Y., Hyeon S.J., Lee D.S., Ryu H. (2017). Increased acetylation of Peroxiredoxin1 by HDAC6 inhibition leads to recovery of Abeta-induced impaired axonal transport. Mol. Neurodegener..

[11] Parmigiani R.B., Xu W.S., Venta-Perez G., Erdjument-Bromage H., Yaneva M., Tempst P., Marks P.A. (2008). HDAC6 is a specific deacetylase of peroxiredoxins and is involved in redox regulation. Proc. Natl. Acad. Sci. USA.

[12] Simoes-Pires C., Zwick V., Nurisso A., Schenker E., Carrupt P.A., Cuendet M. (2013). HDAC6 as a target for neurodegenerative diseases: What makes it different from the other HDACs?. Mol. Neurodegener..

[13] Ferguson B.S., McKinsey T.A. (2015). Non-sirtuin histone deacetylases in the control of cardiac aging. J. Mol. Cell. Cardiol..

[14] Li T., Zhang C., Hassan S., Liu X., Song F., Chen K., Zhang W., Yang J. (2018). Histone deacetylase 6 in cancer. J. Hematol. Oncol..

[15] Brijmohan A.S., Batchu S.N., Majumder S., Alghamdi T.A., Thieme K., McGaugh S., Liu Y., Advani S.L., Bowskill B.B., Kabir M.G. (2018). HDAC6 inhibition promotes transcription factor EB activation and is protective in experimental kidney disease. Front. Pharmacol..

[16] Leng Y., Wu Y., Lei S., Zhou B., Qiu Z., Wang K., Xia Z. (2018). Inhibition of HDAC6 activity alleviates myocardial ischemia/reperfusion injury in diabetic rats: Potential role of peroxiredoxin 1 acetylation and redox regulation. Oxidative Med. Cell. Longev..

[17] Leyk J., Daly C., Janssen-Bienhold U., Kennedy B.N., Richter-Landsberg C. (2017). HDAC6 inhibition by tubastatin A is protective against oxidative stress in a photoreceptor cell line and restores visual function in a zebrafish model of inherited blindness. Cell Death Dis..

[18] Yuan H., Li H., Yu P., Fan Q., Zhang X., Huang W., Shen J., Cui Y., Zhou W. (2018). Involvement of HDAC6 in ischaemia and reperfusion-induced rat retinal injury. BMC Ophthalmol..

[19] Semeraro F., Morescalchi F., Cancarini A., Russo A., Rezzola S., Costagliola C. (2019). Diabetic retinopathy, a vascular and inflammatory disease: Therapeutic implications. Diabetes Metab..

[20] Shin E.S., Sorenson C.M., Sheibani N. (2014). Diabetes and retinal vascular dysfunction. J. Ophthalmic Vis. Res..

[21] Kowluru R.A., Chan P.S. (2007). Oxidative stress and diabetic retinopathy. Exp. Diabetes Res..

[22] Francisqueti-Ferron F.V., Ferron A.J.T., Garcia J.L., Silva C., Costa M.R., Gregolin C.S., Moreto F., Ferreira A.L.A., Minatel I.O., Correa C.R. (2019). Basic concepts on the role of nuclear factor erythroid-derived 2-like 2 (Nrf2) in age-related diseases. Int. J. Mol. Sci..

[23] Zhong Q., Kowluru R.A. (2010). Role of histone acetylation in the development of diabetic retinopathy and the metabolic memory phenomenon. J. Cell. Biochem..

[24] Cai X., Li J., Wang M., She M., Tang Y., Li J., Li H., Hui H. (2017). GLP-1 treatment improves diabetic retinopathy by alleviating autophagy through GLP-1R-ERK1/2-HDAC6 signaling pathway. Int. J. Med. Sci..

[25] Friedrich M., Gerbeth L., Gerling M., Rosenthal R., Steiger K., Weidinger C., Keye J., Wu H., Schmidt F., Weichert W. (2019). HDAC inhibitors promote intestinal epithelial regeneration via autocrine TGFbeta1 signalling in inflammation. Mucosal Immunol..

[26] Borgas D., Chambers E., Newton J., Ko J., Rivera S., Rounds S., Lu Q. (2016). Cigarette smoke disrupted lung endothelial barrier integrity and increased susceptibility to acute lung injury via histone deacetylase 6. Am. J. Respir. Cell Mol. Biol..

[27] Lu Q., Sakhatskyy P., Grinnell K., Newton J., Ortiz M., Wang Y., Sanchez-Esteban J., Harrington E.O., Rounds S. (2011). Cigarette smoke causes lung vascular barrier dysfunction via oxidative stress-mediated inhibition of RhoA and focal adhesion kinase. Am. J. Physiol. Lung Cell. Mol. Physiol..

[28] Siwak M., Maslankiewicz M., Nowak-Zdunczyk A., Rozpedek W., Wojtczak R., Szymanek K., Szaflik M., Szaflik J., Szaflik J.P., Majsterek I. (2018). The relationship between HDAC6, CXCR3, and SIRT1 genes expression levels with progression of primary open-angle glaucoma. Ophthalmic Genet..

[29] Bai J., Lei Y., An G.L., He L. (2015). Down-regulation of deacetylase HDAC6 inhibits the melanoma cell line A375.S2 growth through ROS-dependent mitochondrial pathway. PLoS ONE.

[30] Yang Q., Li S., Zhou Z., Fu M., Yang X., Hao K., Liu Y. (2020). HDAC6 inhibitor Cay10603 inhibits high glucose-induced oxidative stress, inflammation and apoptosis in retinal pigment epithelial cells via regulating NF-kappaB and NLRP3 inflammasome pathway. Gen. Physiol. Biophys..

[31] Guedes-Dias P., Oliveira J.M. (2013). Lysine deacetylases and mitochondrial dynamics in neurodegeneration. Biochim. Biophys. Acta.

[32] Yan J. (2014). Interplay between HDAC6 and its interacting partners: Essential roles in the aggresome-autophagy pathway and neurodegenerative diseases. DNA Cell Biol..

