# Supplementary material for: Inhibition of HDAC6 Attenuates Diabetes-Induced Retinal Redox Imbalance and Microangiopathy
Source: Antioxidants (Basel). 2020 Jul 9;9(7):599. doi: 10.3390/antiox9070599 (PMC7402090; doi:10.3390/antiox9070599)
Supplement: Supplementary file 1 [file antioxidants-09-00599-s001.pdf]

Supplementary data

Table S1. Demographics and clinical history of human *postmortem* retinal donors.

**Donors**	**Experimental Group**	**Age**	**Sex**	**Significant Medical Conditions**	**Ocular History**
P-1	Control/non-diabetic	51	M	HTN, non-invasive eye surgery	
P-2	Control/non-diabetic	38	M		
P-3	Control/non-diabetic	52	M	HTN, high cholesterol	
P-4	Control/non-diabetic	52	M		
P-5	Control/non-diabetic	56	F		
P-6	Control/non-diabetic	56	M	HTN, lupus	
P-7	Control/non-diabetic	48	F	Obesity, CAD	
P-8	Control/non-diabetic	68	M	Coronary artery bypass graft, HTN	
P-9	Diabetic	62	F	Dialysis, HTN, stroke	DR
P-10	Diabetic	60	F	Obesity	DR, laser OS
P-11	Diabetic	67	M	IDDM 10 yrs, toe amputation, dialysis	DR, laser OS
P-12	Diabetic	71	M	IDDM, dialysis, Parkinson's Disease, altered mental state	DR, glaucoma
P-13	Diabetic	57	F	ESRD, IDDM 10yrs, obesity	DR
P-14	Diabetic	49	M	Leg amputation	DR
P-15	Diabetic	52	M	Dialysis	DR
P-16	Diabetic	68	F	Alzheimer's, right lung mass	DR

CAD, coronary artery disease
DR, diabetic retinopathy
HTN, hypertension
IDDM, insulin-dependent diabetes mellitus
NDDM, noninsulin-dependent diabetes mellitus
ESRD, end-stage renal disease
OS, oculus sinister (left eye)

Table S2. Biochemical parameters measured in the experimental rat groups.

Experimental Groups	Body weight (g)	Blood Glucose (mg/dl)	HbA1c (mmol/mol)	ALT (U/L)	AST (U/L)
**Control**	**298±5.0**	**87.6±6.9**	**4.1±0.1**	**56±6**	**115±5.6**
**DB**	**217.8±17.8****	**492±76.4****	**9±0.6***	**101±13.8***	**234±16.9***
**DB + TS**	**251.2±14.3#**	**447.8±67.1**	**7.8±0.5**	**79.6±9.9#**	**118.6±29#**

*p<0.001 and **p<0.0001 vs. control and #p<0.05 vs. DB; n=6

HbA1c, Hemoglobin A1c
ALT , Alanine aminotransferase
AST, Aspartate aminotransferase

Table S3. Primer sequences used in the study.

Genes	Forward	Reverse
Rattus norvegicus		
**HDAC6**	GGA AAA GGT CGC CAG AAA CTT	GGC CGG TTG AGG TCA TAG TT
**GCLC**	GCCGTCTTACAGGGGATGTT	ACGCCTTCCTTCCCATTGAT
**GCLM**	GTGGGCACAGGTAAAACCCAA	ACTTGCCTCAGAGAGCAGTTC
**NQO 1**	TGGCCAATTCAGAGTGGCATT	AGAGTGGTGACTCCTCCCAG
**HO-1**	CTGCTAGCCTGGTTCAAGATACT	TAAATTCCCACTGCCACGGT
**HPRT-1**	TGGATACAGGCCAGACTTTGT	TTGCCGCTGTCTTTTAGGCT
Homo sapiens		
**HDAC6**	GGAAAAGGTCGCCAGAAACTT	GGCCGGTTGAGGTCATAGTT
**18s**	GGCCCTGTAATTGGAATGAGTC	CCAAGATCCAACTACGAGCTT

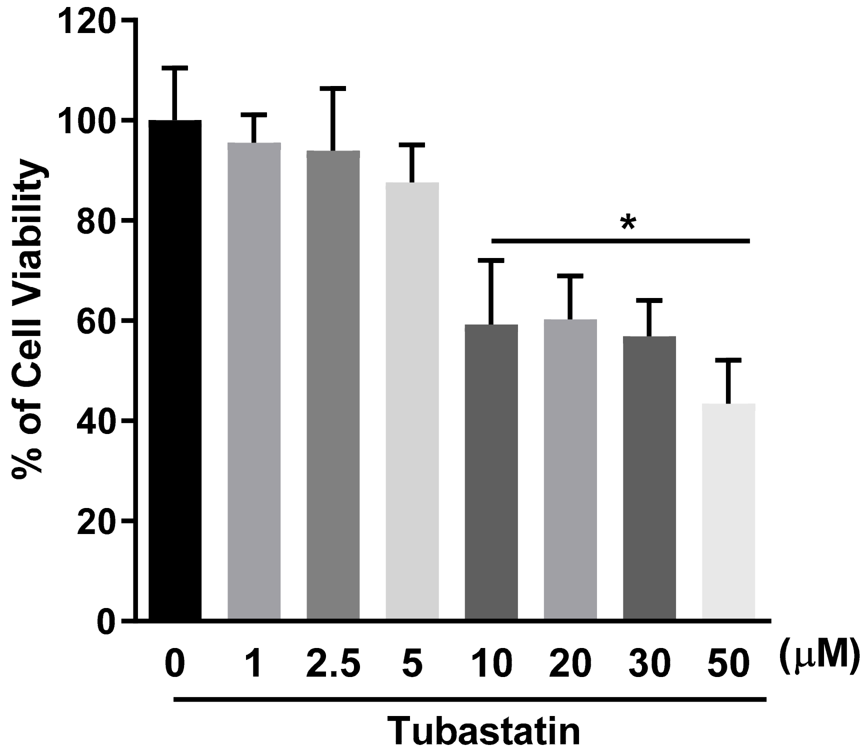

Figure S1. MTT Assay was performed to assess viability of HuREC cells in response to different concentrations of Tubastatin A (1-50 μM). Values are mean ± SEM for n=6. *p<0.05 vs. 0 μM.
